# Histamine, Metabolic Remodelling and Angiogenesis: A Systems Level Approach †

**DOI:** 10.3390/biom11030415

**Published:** 2021-03-11

**Authors:** Aurelio A. Moya-García, Almudena Pino-Ángeles, Francisca Sánchez-Jiménez, José Luis Urdiales, Miguel Ángel Medina

**Affiliations:** 1 Departamento de Biología Molecular y Bioquímica, Universidad de Málaga, 29071 Málaga, Spain; amoyag@uma.es (A.A.M.-G.); medina@uma.es (M.Á.M.); 2 Instituto de Investigación Biomédica de Málaga (IBIMA), 29010 Málaga, Spain; 3 Unidad de Lípidos y Arteriosclerosis, Servicio de Medicina Interna, Hospital Universitario Reina Sofia, Instituto Maimonides de Investigación Biomédica de Córdoba (IMIBIC), Universidad de Córdoba, 14004 Córdoba, Spain; almudena.pino@imibic.org; 4 Centro de Investigación Biomédica en Red de Fisiopatología de la Obesidad y la Nutrición (CIBEROBN), Instituto de Salud Carlos III, 14004 Córdoba, Spain; 5 Centro de Investigación Biomédica en Red de Enfermedades Raras (CIBERER), Instituto de Salud Carlos III, 29010 Málaga, Spain; kikafriky@gmail.com

**Keywords:** metabolic remodelling, angiogenesis, systems biology

## Abstract

Histamine is a highly pleiotropic biogenic amine involved in key physiological processes including neurotransmission, immune response, nutrition, and cell growth and differentiation. Its effects, sometimes contradictory, are mediated by at least four different G-protein coupled receptors, which expression and signalling pathways are tissue-specific. Histamine metabolism conforms a very complex network that connect many metabolic processes important for homeostasis, including nitrogen and energy metabolism. This review brings together and analyses the current information on the relationships of the “histamine system” with other important metabolic modules in human physiology, aiming to bridge current information gaps. In this regard, the molecular characterization of the role of histamine in the modulation of angiogenesis-mediated processes, such as cancer, makes a promising research field for future biomedical advances.

## 1. Histamine Metabolism and its Connections to Other Metabolic Modules

Histamine (2-(1H-Imidazol-4-yl) ethanamine) is the product of the alpha decarboxylation of the essential amino acid histidine (2-Amino-3-(1H-imidazol-4-yl) propanoic acid) by the enzyme histidine decarboxylase (HDC). Histidine is required in early embryonic stages and during childhood as an exogenous source of histamine, as well as in adults with certain health conditions, such as those caused by the impairment of nitrogen metabolism (i.e., malnutrition, cachexia, hepatic or renal problems, among others). Its synthetic pathway is well known in bacteria [1], hence both dietary proteins and microbiota catalytic activity are the main source for histidine availability. Histidine is also an antioxidant precursor, and its deficiency translates into low levels of histamine, which critically alters the normal functioning of the nervous and immune systems [2,3,4]. The imidazole ring in histidine acts as electron acceptor/donor in many enzymatic basic-acid reactions, and it is essential in electron transfer systems, oxygen transport, and the function of Zn^2+^-dependent enzymes and transcription factors, thanks to its ability to form complexes with bivalent metal ions, such as Fe^2+^, Cu^2+^, Co^2+^, Ni^2+^, Cd^2+^, and Zn^2+^. See the review by Holeček [5] for further information.

**Figure 1:**
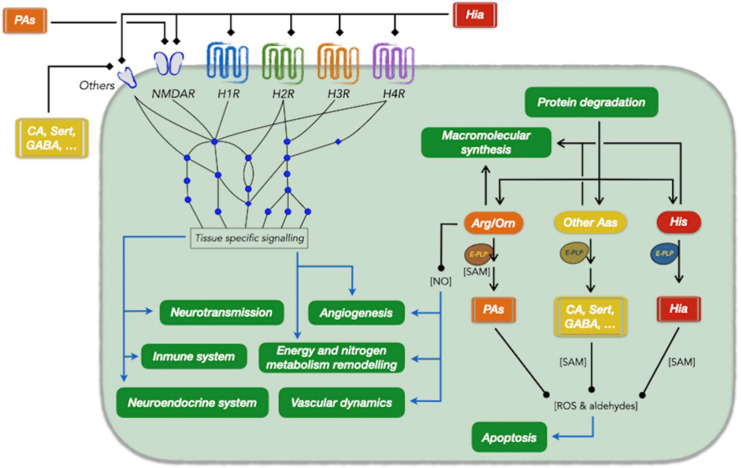
Relationships between histamine and other amines with different physiological and cellular processes. The processes are depicted in green boxes. Abbreviations (by alphabetical order): Aas, amino acids; Arg/Orn, arginine/ornithine; CA, catecholamines; E-PLP, pyridoxal phosphate dependent enzyme; GABA, gamma aminobutyric acid; H1R, histamine receptor 1; H2R, histamine receptor 2; H3R, histamine receptor 3; H4R, histamine receptor 4; Hia, histamine; His, histidine; NMDAR, N-Methyl-D-aspartic acid receptor; NO, nitric oxide; PAs, polyamines; ROS, reactive oxygen species; SAM, S-adenosyl methionine; Sert, serotonin.

Histamine is not only a key player in allergic reactions but also in vascular permeability, circadian cycle regulation and other neurological and gastric functions, epithelium proliferation, immune cell differentiation, other cardiovascular functions, angiogenesis, and neoplastic progression (Figure 1). Histamine can modify the proteome by degradation-derived reactive oxygen species (ROS) that oxidize Cys and Tyr residues, promoting functional alterations that depend on the protein and the physiological status of the cell. Proteins involved in cytoskeleton organization, muscle contraction, inflammation, and cell signalling [6], including G protein-coupled receptors (GPCRs) [7] can also be modified covalently by transglutaminase 2 (TG2), which uses histamine as a substrate [8]. Experiments with HDC KO mice have shown that histamine plays a central role in multiple human pathologies [9,10], although it is still unknown how the elements in the metabolism of histamine are linked to those involved in the development of these conditions.

At the metabolic level, L-His and histamine are key molecules of nitrogen homeostasis. Histamine metabolism shares metabolites (pyridoxal 5-phosphate, PLP, and S-adenosine methionine, SAM), enzymatic activities (diamine oxidase, DAO, monoamine oxidase, MAO, aldehyde dehydrogenase, and transglutaminase 2) and membrane transporters with the metabolic pathways of other biogenic amines (the diamine putrescine, the polyamines (PA) spermidine and spermine, dopamine and serotonin), and all of them depend on protein intake and endogenous protein synthesis rates (Figure 1). Multiple evidence show that these metabolic pathways also share regulatory mechanisms. Antagonistic time courses of histamine and PA metabolisms have been observed in several pathophysiological scenarios such as in murine mast cells differentiation and basophilic leukemia cells [11,12,13,14]. This evidence has been recently reviewed in the context of different pathophysiological scenarios [15,16]. The role of biogenic amines in pathologies affecting the neurological and immune systems, mucosa and epithelium renewal and permeability, and fertility (i.e., neurodegenerative diseases, mental retardation, psychiatric disorders, inflammatory reactions, food allergies, cancer progression and several rare diseases) should be considered to fully characterize these diseases. We will discuss several links between these metabolic and pathophysiological networks in the context of metabolic remodelling and angiogenesis-related diseases.

Histamine metabolism is also connected to enzymes of the vitamin B6-dependent family, which use PLP as their cofactor. Among these, there are decarboxylases (including those responsible of other biogenic amines such as dopamine, serotonin, GABA, among others), amino transferases, and other transferases, synthases and lyases [17]. PLP-dependent enzymes are key participants in nitrogen metabolism, including nitrogen homeostasis, the synthesis of neurotransmitters, hormones and neuroendocrine mediators, folate and 1-carbon metabolism, protein and polyamine synthesis, carbohydrate and lipid metabolism, mitochondrial function and erythropoiesis [15]. Hence, vitamin B6 deficit could affect histamine synthesis, as well as other catalytic products of PLP-dependent enzymes, therefore, histamine deficit is a factor to be considered to explain diverse disease phenotypes [18].

Histamine binds to four known G protein-coupled receptors (GPCRs), H1 to H4 receptors, that modulate different signalling pathways [19]. The physiological effects of histamine are dependent on the receptor expressed by each cell type in each physiological context, oftentimes inducing opposite effects on different cell types or cellular status. These facts add further complexity to the analysis and discussion of experimental results. The signalling pathways activated by the four histamine receptors have been reviewed in several recent publications [15,19] and are also summarized in Table 1.

The rate-limiting step in histamine synthesis is the decarboxylation reaction by HDC, a very instable, short-lived PLP-dependent enzyme homologous to L-aromatic amino acid decarboxylase. In rat stomach, the half-life of HDC activity was 55 min in controls [24]. The maturation of HDC involves trafficking through the endoplasmic reticulum in a process still not fully characterized, and several proteolytic systems, such as proteasome, m-calpain and caspase-9, may be involved in its maturation or degradation [25,26,27,28,29]. The first model of the quaternary structure for the N-terminus of rat HDC (first 512 residues) was obtained by homology modelling and further validated by direct-mutagenesis experiments [27,30,31]. The enzyme kinetics and molecular properties were extensively reviewed in 2005 [32]. More recently, the structure of a stable double mutant of the human HDC in complex with the inhibitor histidine methyl esther was solved by X-ray crystallography at 1.8 Å resolution [33]. Both the X-ray structure and computational model are rather coherent in terms of global folding. As of today, the structure of the wild type enzyme has not been solved by any experimental method. A recombinant human fragment (512-N-terminus) of the native rat homodimer—with the maximum activity assayed in vitro [31]—is very instable and exhibit an extremely low Vmax [25,34], which suggest that an uncharacterized element could be stabilizing the native dimer conformation in vivo. In addition, further evidence indicates that HDC polypeptide length and the location where it performs is activity in the cell are key factors in the physiology of histamine-producing cells. In fact, the overexpression of an active recombinant version of rat HDC in a human cell-type unable to store histamine, such as HEK or COS cells, alters their proteome with lethal consequences in cell cultures, including increased expression of apoptotic caspases and α-synuclein in the case of HK cells [35].

HDC expression is limited to a few cell types: histamine-storing cells (i.e., mast cells, basophils, parietal gastric cells and neurons) and cells that synthetize histamine but cannot store it in specialized vesicles—that is other immune cells such as macrophages, eosinophils and platelets, and several types of cancer cells) [4]. By analysing the HDC expression in different tissues, we observed that the major histamine-producing cells (mast cells, gastric enterochromaffin-like cells and histaminergic neurons) are surrounded by other cell types, promoting a tight relationship with physiological relevance [36,37,38]. These cells maintain a complex communication that depends on the expression of the histamine receptors, as well as on the own signalling proteome expressed by the histamine-producing cells. For instance, production and secretion of histamine in mast cells is also regulated by PA [39] as well as by the ornithine decarboxylase antizyme inhibitor 2, which is also an activator of PA synthesis and uptake expressed in mast cells [14,40,41].

Histamine synthesis rate depends on the HDC expression regulation, a process which seems to have a strong cell-specific component. Yatsunami et al. [42] located promoter fragments involved in HDC induction by TPA plus dexamethasone and cAMP plus Ca^2+^ in basophilic cells. HDC regulation in basophilic cells seems to involve several cis-elements such as a TATA-like sequence, a GC box, four CACC boxes, four GATA sequences and 6 leader-binding protein-1 binding motifs, as well as a c-Myb motif, and other positive and negative motifs located in the promoter between positions -497 and -855 in the gene. In ECLC, gastrin is an important HDC inducer [43], involving 3 gastrin-responsive elements. *Helicobacter pylori* infection activates a MEK1-2/ERK1-2 cascade resulting in gastric HDC induction. However, Kruppel-like factor, Ying-yang and SREBP act as repressors of HDC expression [44,45]. Promoter methylation also seems to play an important role in regulation of HDC expression in different cell types, a mechanism that seems to be dependent of specific cell differentiation programs and cell metabolic status [46,47]. In differentiated histamine-producing cells, HDC expression is regulated by different signalling pathways, which coordinates histamine production with its physiological function (immune response, gastric acid secretion, neurotransmission, cancer progression) in differentiated cell types. Current information on the regulatory mechanisms of histamine synthesis in inflammatory and gastric cells has been provided by Wang and collaborators. Nevertheless, knowledge on HDC transcriptional regulation in differentiated human cell types still is an open subject of study with important gaps in tissue-specific information [15,48].

Histamine can be degraded by two different pathways in mammalian cells. On one hand, N-methyl transferase (HNMT) carries out the intracellular N-methylation of the imidazole group, using SAM as the methyl donor [49]. HNMT is a 33kDa monomeric protein, A human recombinant version being solved by X-ray chrystallography, Its activity is sensible to SH-group reagents [50,51]. Its gene is poorly characterized and lacks canonical promoter cis-elements such as TATA and CAAT boxes [52]. The activity links histamine degradation to the availability of SAM, and to the synthesis of polyamines, and folates/methionine recycling [53,54], which is involved in the methylation of proteins and nucleic acids, being therefore relevant for the regulation of gene expression and epigenetics. HNMT is mainly expressed in liver and seems to be the major histamine degradation pathway in the brain [15]. The product N-tele methyl-histamine is a substrate of MAO-B, which produces oxygen peroxide and N-methylimidazole acetaldehyde. MAO-B is a member of the flavin monoamine oxidase family, located in the mitochondrial outer membrane. It is expressed mainly in liver and other tissues in a minor extent [55]. In regulation of its transcription, the complex Sp1/Egr1/CREB, as well as microRNAs miR-1224 and miR-300, seem to be involved [56]. In other tissues, such as the gastrointestinal tract, histamine is oxidized by DAO, a cytosolic membrane-associated protein that produces imidazole acetaldehyde and oxygen peroxide [49]. This enzyme has also be detected in body fluids [57,58]. It is worth mentioning that mucosal mono- and polyamine oxidase activities are distributed complementary to diamine oxidase in digestive tract [59]. Whatever the degradation pathway is, histamine is a source of reactive oxygen species (ROS) that can cause macromolecular damage and aging, especially if the microenvironment is poor in antioxidant molecules. In other cases, reversible protein oxidation by ROS can have beneficial effects for certain cell types; for instance, preconditioning neurons to ischemia [60].

## 2. Histamine and Vessel Dynamics

It is well known that alterations in vessel dynamics and vascular permeability are tightly connected with angiogenesis [61]. As early as 1948, histamine was seen to increase the vascular permeability at the haemato-ocular barrier [62], and just three years later, Miles showed that histamine increases the permeability of skin capillaries in guinea pigs [63]. Several studies published in the following years confirmed and reinforced these findings [64,65,66,67], and shed light into the molecular mechanisms, including the roles of protein kinases, cytosolic calcium, cAMP and actin cytoskeleton [68,69]. Guo et al. showed that histamine causes a transient and reversible disruption of the VE-cadherin/beta-catenin binding during the hyperpermeability response induced by histamine on endothelial cells [70]. The increase in vascular permeability induced by histamine through histamine H1 receptor had a direct effect on vascular permeability for low-density lipoproteins, thus promoting the formation of atherosclerotic lesions [71].

H3 and H4 receptors are also expressed by endothelial cells, so there might be other potential effects of histamine on vascular permeability and vessel dynamics still unknown [72]. The role of histamine in vascular leakage and dysfunction is mediated by RhoA and ROCK [73,74,75]. RhoA activation induced by histamine is associated to a fast Ca^2+^ influx and the breakdown of microvascular endothelial cell barriers [76]. In lymphatic endothelial cells, this movement of Ca^2+^ is mediated by Ca^2+^ release-activated Ca^2+^ channels (CRAC), suggesting that CRAC could be a target for inhibitors able to relieve histamine-triggered vascular leakage [77].

Recently, Grimsey et al. [78] have shown that histamine induce a robust p38 autophosphorylation in endothelial cells, acting through H1R/H2R. This involves the non-canonical TAB1-TAB2/3 dependent pathway, thus promoting endothelial inflammatory responses. This agrees with our previous observations on the anti-inflammatory effects of epigallocatechin gallate (EGCG), an inhibitor of HDC, on mast cells and monocytes [79,80].

## 3. Histamine, Metabolic Reprogramming and Angiogenesis: Pathophysiological Implications

Cell proliferation and differentiation in different pathophysiological scenarios, such as gestation or cancer progression, are the most relevant processes subject to metabolic reprogramming induced by histamine. Only the cells able to adapt their metabolic networks to the pressure posed in such processes can keep its own homeostasis and survive.

### 3.1. Histamine and Angiogenesis in Gestation

Biogenic amines have regulatory roles in the metabolic changes and complex cellular communication events that occur during gestation, i.e., energy and nitrogen metabolism remodelling, adaptation to hypoxia, extracellular matrix remodelling, endothelial and immune cell differentiation, and the production, secretion, and reception of mast cells mediators [12,81,82,83].

Histamine levels modulate embryo-uterine interactions, so either low or excessively high levels might lead to gestational complications [84,85]. Histamine seems to have a regulatory role in trophoblast differentiation—a process that involves the expression of integrin aV-b3 and trophoblastic H1R, which seems to be involved in vascular invasion and placenta neovascularization [86]. Further research suggests that histamine-induced tissues remodelling favours embryo implantation, assisted by several molecular mediators released by infiltrated mast cells [87]. Those infiltrated mast cells present in the placental bed are involved in immune regulatory functions, the regulation of trophoblast invasion, in angiogenesis and in vessel remodelling [88].

### 3.2. Histamine in Cancer

The involvement of histamine in different cancer types has been extensively reviewed [89,90]. Histamine can participate in several of the hallmarks of cancer introduced by Hanahan and Weinberg [91]. Histamine can induce cancer cell proliferation or cell death depending on the receptors expressed by the target cells. This includes autocrine effects, since some cancer cells are also histamine-producing cells (as for instance, malignant mastocytosis [92], breast cancer [93], or gastric cancer [94]), and paracrine effects between cancer cells and the histamine-producing immune cells—including those that produce histamine—in the tumour microenvironment [37]. H1R-elicited signals have been considered mainly as antiproliferative stimuli; the opposite role has been proposed by histamine when acting through H2R, however some controversial results were obtained in vivo [93,95,96]. The latest discovered histamine receptor (H4R) also seems to be an important element for modulation of carcinogenesis and/or cancer progression. Working with breast cancer, H4R agonists reduce several markers of tumour progression in vivo [97]. It is interesting that both tumour and immune cells can express H4R, suggesting that histamine acting through H4R could be a coordinator of the crosstalk between immune and breast cancer cells in vivo. Similar results were obtained for H4R working with melanoma cell lines [98]. The results could indeed have translational potential, but further characterization of the signalling at molecular level is still needed [90]. This complex intercellular interactome must plays very important roles in the development of several hallmarks of cancer different from those related with cancer growth (sustaining proliferative signalling and evading growth suppressors). For instance, histamine enables the tumour to suppress the immune response from the tumour environment and promotes inflammation, leading to invasion, metastasis, and angiogenesis with the participation of cytokines and proteases [99,100].

### 3.3. Histamine and Angiogenesis

A deregulated angiogenesis is casually involved in many diseases, including cancer, ophthalmic diseases, arthritis, psoriasis and almost 200 rare diseases [101,102]. The role of histamine in angiogenesis, was first suggested in 1983 when Fraser and Simpson showed that histamine produced by mast cells induces neovascularization in a chorioallantoic membrane model; however, this induction was not replicated by Barnhill and Ryan who reported negative results with histamine 0.1 mM [103,104]. Further research supported the indirect inductive effect of histamine on angiogenesis [105,106], mediated by H1R and H2R [107]. The effect of histamine and serotonin in angiogenesis is biphasic: micromolar concentrations of histamine give rise to a quick pro-angiogenic response via TR3/NUR77, but induce a negative feed-back loop after 10 days by upregulating the potent anti-angiogenic endogenous compound thrombospondin-1 [108].

Endogenous histamine has a dual role (activation/inhibition) in the regulation of angiogenesis [109]. The effects of histamine produced by mast cells have been reviewed in depth [110,111,112] and they are dependent on the activity of several transcription factors, including NR4A1, MYCN and RCAN1, [113]. The role of H1R and H2R is also well established, where the synergistic effect of histamine and bFGF mediated by H1R increases VEGF levels and thus induces angiogenesis [114]. Furthermore, the H2R antagonist cimetidine inhibits angiogenesis [115].

## 4. A Systems Biology Approach to Histamine as a Modulator of Metabolic Reprogramming

The large amount of evidence gathered from traditional biochemical and cellular biology experiments together with the latest generalized use of -omics techniques have revealed that molecular functions and phenotypes can be no longer understood as isolated events. The intricate reality of human pathophysiology has prompted the use of systemic approaches to study the intertwined molecular mechanisms underlying health and disease [116]. Contrary to classic pharmacology approaches, the tools in Systems Biology allow us to move past the traditional paradigm of “one gene–one protein (target)–one drug”. Systems Biology explores the complex networks of molecular interactions and signaling controlling the biological functions, and by extension, Systems Pharmacology will assess what elements in these networks need to be modulated to regulate precisely and effectively the outcome of such functions. Systems Biology is then essential in the development of successful personalized therapies, especially in multifactorial conditions that have large variability between individuals.

In previous sections, histamine has been shown to have a potential role in the metabolic remodelling of cancer cells. Through histamine receptors, histamine can influence several hallmarks of cancer, and participate in important processes underlying tumour progression and metastasis, such as angiogenesis and inflammation. However, the many molecular pathways by which histamine operates often branch and crosstalk, making up an intricate network of molecular interactions. The complexity of such interactions makes the characterisation of histamine pathways a challenging task that can be tackled by Systems biology approaches.

**Figure 2:**
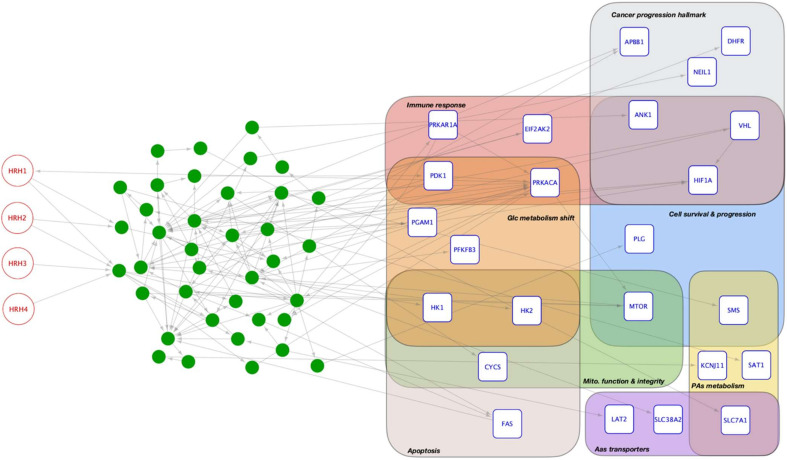
Network of signaling interactions between the histamine receptors and the genes involved in reprogramming cancer metabolism. Abbreviations used as in Table 2.

We have modelled the network of signalling interactions between the histamine receptors and the key metabolic modulators in the tumour microenvironment (compiled and reviewed in [117]) to gain insight of the potential role of histamine in tumour metabolic reprogramming. We compiled the signalling interactions from well-regarded data repositories: the Atlas of Cancer Signalling Networks [118], the Cancer Cell Map [119], PhosphoSite [120], the Signalling Network Open Resource [121], the Human Cancer Signalling Network [122], and OmniPath [123]; from which we modelled the network of signalling interactions between the histamine receptors and the genes involved in reprogramming cancer metabolism, so we obtained a general view of how histamine participates in reprogramming tumour metabolism. Table 2 and Figure 2 show that histamine receptors are connected through signalling interactions with genes involved in disparate signalling pathways that regulate the cellular metabolism, glycolysis, the TCA cycle and oxidative phosphorylation, the synthesis of lipids and nucleic acids, and in particular, the metabolism of polyamines and amino acids.

As expected, some of the targets of signalling interactions from histamine receptors are involved in immune response: ANK1, EIF2AK2, HIF1A, PDK1, PRKACA, PRKAR1A and VHL. We postulate that the signalling exerted by histamine would be relevant in the crosstalk between proliferating cancer cells and immune cells present in the tumour microenvironment. We observe that histamine can influence the shift from aerobic to anaerobic glucose metabolism that cancer cells deploy in response to proliferation stimuli [124], by influencing key regulators of glucose metabolism such as HK1, HK2, PDK1, PRKACA and PFKFB3. Histamine could mediate signalling effects on the mitochondria function and integrity through CYCS, hexokinase isozymes, and mTOR.

The metabolism of histamine and the biogenic polyamines are related to each other in some cell types. We have modelled their interactions—including the crosstalk with sulphur metabolism—that provided deeper insights on the connections of polyamines with energy metabolism [47,53,125]. In our network models, it can be observed how histamine connects with polyamines metabolism through spermine synthase (SMS) and spermidine/spermine acetyl transferase (SAT1) and the cationic amino acid transporter (SLC7A1), which have been described previously in cancer cell models [16,126].

Furthermore, our network analysis supports previous evidence that histamine can sway the metabolism of both polyamines and amino acids, including those essential for cancer cell growth such as glutamine, arginine, methionine and ornithine [127]. However, the signalling effect of histamine on polyamines and amino acid metabolism comes up mainly by histamine influencing polyamines and amino acids transporters (see Table 2). This observation remarks the importance of the metabolic compartments for understanding biochemical and physiological processes [128].

Our histamine signalling network provides further details on the ambiguous role histamine plays on regulating both cell proliferation and cell death. On the one hand, histamine participates in the regulation of apoptosis acting upon CYCS, FAS, and hexokinases. On the other hand, histamine can influence cell survival and cell proliferation pathways by way of key elements such as mTOR, HIF1A, SMS, and VHL, together with elements of cancer progression hallmarks—ANK1, involved in cytoskeleton remodelling, DHFR in genome methylation, NEIL1 in genome edition, APBB1 and VHL in chromatin covalent modification, and the remarkable role of HIF1A as coordinator of different cancer hallmarks [129].

## 5. Concluding Remarks and Future Prospects

The complex system we termed the histamine influence network has several remarkable features. Its basic metabolism (synthesis and degradation) consists of simple pathways composed by a few reactions. However, the expression of key enzymes is cell type specific, and their regulation is not well characterised in the different cell types. These metabolic reactions share elements with other biochemical pathways and can therefore take part in processes such as SAM-dependent methylation of amino acids and amines, and posttranslational protein covalent modification, thus expanding the histamine metabolic network.

Besides synthesis and degradation, metabolism also includes intercellular signalling, adding further complexity to the histamine network. Since histamine receptors are expressed in different cell types, activate different G proteins and show different affinity for histamine (Table 1), histamine can conform an extremely complex metabolic network that modulates the key physiological processes at the organism level. The greater advances in biomedicine and pharmacology regarding histamine-mediated pathologies focus almost exclusively on allergic and immune reactions, neurotransmission and digestion. However, the many data and information gaps that remain on this complex system hampers the development of efficient intervention strategies in many other diseases.

In this work, we have outlined the current perspective on different layers of the network where the role of histamine is clear, with our focus on unveiling further details on cell proliferation, tissue growth and cancer progression.

Tumour development requires distinct metabolic phenotypes to support their proliferation and progression, especially in both nitrogen and energy metabolism. Cell signalling is fundamental to understand this metabolic reprogramming, but a full view combining the known individual signalling pathways is still missing. We have provided a glance of how histamine is involved in the coordination of some signalling processes. That is, our network analysis brings an interesting starting point to get further insights in the reprogramming of the metabolism needed for the processes we have discussed in this review.

We propose that Systems Biology approaches are essential to fill the gaps of information about histamine signalling in different pathophysiological scenarios, and to assist the diagnosis and to locate intervention targets of multiple human syndromes and diseases [130]. Our work provides a general picture on how histamine can influence angiogenesis and cancer metabolic reprogramming, though the actual role of histamine will depend on its local concentrations, and the sets of these genes expressed in each cell type involved—in particular the histamine receptors. Experimental approaches using co-cultures or genetic modified organisms, together with transcriptomic, proteomic, metabolomics, and biochemical and biophysical analyses, will also be essential and complementary to understand the effects of histamine in different cell types and physiological conditions.

## Figures and Tables

**Table 1:** Molecular and functional properties of human histamine receptors.

	H1 Receptor	H2 Receptor	H3 Receptor	H4 Receptor
HGNC	HRH1	HRH2	HRH3	HRH4
UniprotKB	P35367	P25021	Q9Y5N1	Q9H3N8
Mass (kDa)	55.7	40.1–44.5 (2 isoforms)	36.4–49.6 (7 isoforms)	34.5–44.5 (2 isoforms)
Binding affinity	Low (2.5 × 10^−5^ M)	Low (7.9 × 10^−6^ M)	High (6.3 × 10^−9^ M)	High (7.9 × 10^−9^ M)
Cell/tissue expression	Ubiquitous, brain, smooth muscle, epithelial and endothelial cells, immune cells, hepatocytes and chondrocytes	Ubiquitous, gastric-mucosa parietal cells, smooth muscle, heart, epithelial and endothelial cells, immune cells, hepatocytes and chondrocytes	High expression on histaminergic neurons	High expression on bone marrow and peripheral hematopoietic cells
G_α_ protein coupling	G_α__q/11_	G_α__s_	G_α__i/o_	G_α__i/o_
Signalling pathways	PLC activation, increase of Ca^2+^, PKC activation, NOS activation, increase of cGMP, cAMP accumulation (via G_βγ_ subunits)	PKA activation, increase of cAMP, PLC activation, increase of Ca^2+^	Decrease of cAMP, inhibition of Ca^2+^ channels, stimulation of MAP kinase phosphorylation	Decrease of cAMP, inhibition of Ca^2+^ channels, stimulation of MAP kinase phosphorylation
Primary functions	Immediate allergic response. Inflammatory response	Gastric acid secretion. Suppression of immune cells. Inflammatory response	Regulation of arousal and cognition. Control of inflammatory response	Allergic and inflammatory responses. Immune cell chemotaxis

Data from references [15,19,20,21,22,23].

**Table 2:** A synthesis of biochemical characteristics of metabolic remodelling-related targets modified by histamine through H1-H4 receptors.

Protein	HGNC	UniprotKB	Biological Function	Metabolism Remodelling
hexokinase 1	HK1	P19367	Key glycolytic enzyme responsible of hexose phosphorylation, also involved in release of mitochondrial pro-apoptosis elements.	
hexokinase 2	HK2	P52789	Key glycolytic enzyme responsible of hexose phosphorylation	Glycolysis
6-phosphofructo-2-kinase/fructose-2,6-biphosphatase 3	PFKFB3	Q16875	Key enzyme for glycolysis regulation. Proposed as a marker to distinguish between induced-pluripotent stem cells and cancer stem cells. Its expression is modified by hypoxia	
pyruvate dehydrogenase kinase 1	PDK1	O15530	It activates by phosphorylation targets such as AKT1, PRKACA, involved in glucose and nitrogen uptake y storage. It can inhibit TGF-β signalling, as well as activate NF-kB in macrophages and calcium movements in mast cells. Regulator of key nutrient receptor in thymocytes, and essential for mobility of vascular endothelial cells.	TCA cycle
cytochrome c	CYCS	P99999	Electron carrier protein that plays a role in the mitochondrial-associated mechanism of apoptosis	OXPHOS
phosphoglycerate mutase 1	PGAM1	P18669	Glycolytic enzyme described as a promising target for diagnosis and therapy of cancer	Pentose phosphate pathway
SLC7A8 amino acid transporter light chain, L system	LAT2	Q9UHI5	Neutral amino acid cytosolic exchanger. It is involved in glutamine-dependent mTOR activation to promote glycolysis in cancer cells.	Amino acid metabolism
solute carrier family 38 member 2	SLC38A2	Q96QD8	It has glutamine as a ligand, and is involved in cellular response to starvation, regulation of gene expression and splicing, and cellular response to stress.	
solute carrier family 7 member 1	SLC7A1	P30825	It accepts L-Arg, L-ornithine, L- His and L- Lys as substrates.	
potassium inwardly rectifying channel subfamily J member 11	KCNJ11	Q14654	It acts as a transmembrane transport system and an ankyrin-binding protein. It is Involved in cardiac muscle function, ischemia response and glucose homeostasis.	Polyamine metabolism
spermidine/spermine N1-acetyltransferase 1	SAT1	P21673	Key enzyme for polyamine degradation. Highly regulated.	
spermine synthase	SMS	P52788	Enzyme responsible of spermine synthesis from spermidine and decarboxylated S-adenosylmethionine. Diminished activity is related to Snyder-Robinson syndrome.	
Fas cell surface death receptor	FAS	P25445	Key element for extrinsic apoptosis pathway. Related to regulation of immune response.	Lipid synthesis
amyloid beta precursor protein binding family B member 1	APBB1	PO00213	Transcription coregulator related to histone postranslational modifications, and regulation of many key elements for cell division and apoptosis.	Nucleic acid metabolism
dihydrofolate reductase	DHFR	P00374	Key element for biomolecular methylations important for DNA synthesis and gene expression, among many other processes.	
ankyrin 1	ANK1	P16157	Structural protein related to cytoskeletal remodelling, and organelle organization.	Metabolic-signaling pathways
eukaryotic translation initiation factor 2 alpha kinase 2	EIF2AK2	P19525	Protein kinase acting as an inhibitor of viral infection via the integrated stress response. Also involved in regulation of apoptosis and cell proliferation, and inflammatory response.	
hypoxia inducible factor 1 subunit alpha	HIF1A	Q16665	Under hypoxia, it activates a plethora of genes, involved in embryonic vascularization and tumour angiogenesis. Also related to response to virus infections, including SARS-CoV-2.	
mechanistic target of rapamycin kinase	MTOR	P42345	Central regulator of cellular metabolism, growth and survival in response to hormones, growth factors, nutrients, energy and stress signals.	
Endonuclease 8-like 1	NEIL1	Q96F14	Involved in base excision repair of DNA damaged by oxidation or by mutagenic agents.	
Plasminogen	PLG	P00747	Plasmin precursor. Plasmin acts as a proteolytic factor in a variety of other processes including ovulation, embryonic development, tissue remodelling, tumour invasion, and inflammation.	
protein kinase cAMP-activated catalytic subunit alpha	PRKACA	P17612	This kinase is involved in many processes related to fuel (glucose and lipid) metabolism, cell differentiation of different cell-types, and immune cells responses, including inflammation. When activated inhibits the antiproliferative and antiinvasive effect of difluoromethylornithine (an inhibitor of polyamine synthesis).	
protein kinase cAMP-dependent type I regulatory subunit alpha	PRKAR1A	P10644	Subunit responsible of the regulation of cAMP-dependent protein kinase, whose properties are briefly described above.	
von Hippel-Lindau tumor suppressor	VHL	P40337	Involved in the ubiquitination and subsequent proteasomal degradation of proteins. It is involved in transcriptional repression through interactions with H1F1A, HIF1AN and histone deacetylases.	

Data from UniProt Knowledgebase and reference [117].
